# Reduction of myocardial necrosis using “CLOSE” protocol during pulmonary vein isolation—Preliminary results from ACTIVE‐AF trial

**DOI:** 10.1002/joa3.12424

**Published:** 2020-09-02

**Authors:** Michał Peller, Piotr Lodziński, Paweł Balsam, Kacper Maciejewski, Krzysztof Ozierański, Bartosz Krzowski, Grzegorz Opolski

**Affiliations:** ^1^ 1st. Department of Cardiology Medical University of Warsaw Warsaw Poland

**Keywords:** atrial fibrillation, catheter ablation myocardial necrosis, pulmonary vein isolation, troponin I

## Abstract

**Background:**

New protocols of pulmonary veins isolation (PVI) result in easier and more efficient procedure performance. Ablation index (AI) is the novel tool which helps to achieve transmural lesions during catheter ablation. However, benefit of this protocol in the reduction of myocardial injury is still not known.

**Purpose:**

The aim of the study was to compare myocardial injury during catheter ablation using standard and AI protocol.

**Methods:**

To the analysis we included 24 patients with paroxysmal atrial fibrillation, who underwent radiofrequency catheter PVI using CARTO system (Biosense Webster, Inc). In all patients cardiac troponin I (cTnI) levels were assessed before and 24 hours after the procedure. In 12 patients PVI was performed using continuous applications (dragging technique) and in 12 patients during PVI ablation AI protocol was implemented. To unify analyzed groups, we excluded patients with additional ablation lines (including line separating ipsilateral pulmonary veins).

**Results:**

In analyzed group mean age was 59.3 ± 7.7 years and 18 (75%) patients were male. There were no differences in the clinical characteristic between both subgroups. Trend in shorter total x‐ray time was observed in AI group compared with dragging group (8.6 ± 5.4 vs. 5.3 ± 3.2 min.; *P* = .093) with no differences in total procedure time (146.3 ± 28.9 vs. 153.2 ± 37.1 min.; *P* = .616). Twenty‐four hours after the PVI procedure cTnI levels were significantly lower in AI group than in dragging group (1.984 ± 0.644 vs. 3.369 ± 1.818 ng/mL; *P* = .026), with no difference in mean baseline cTnI levels (0.004 ± 0.006 vs. 0.015 ± 0.032 ng/mL; *P* = .304).

**Conclusion:**

Presented study revealed that compared with standard, continuous applications, AI protocol implementation results in reduction of myocardial injury during catheter PVI in patients with paroxysmal atrial fibrillation.

## Graphical abstract

Ablation Index (AI) is the novel tool which helps to achieve transmural lesions during catheter ablation. Presented study revealed that compared with standard, continuous applications, AI protocol implementation results in reduction of myocardial injury during catheter PVI in patients with paroxysmal atrial fibrillation.

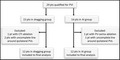

## 1 INTRODUCTION

According to current guidelines, pulmonary vein isolation (PVI) should be performed in symptomatic patients in whom we use a rhythm control strategy, especially in the absence of antiarrhythmic drug efficacy.^1^ Despite of the lack of mortality reduction with PVI compared to antiarrhythmic drugs, invasive treatment significantly reduces the incidence of arrhythmias.^2^ In recent years we have observed the development of invasive techniques. However, the frequency of atrial fibrillation (AF) recurrence after PVI treatment is still relatively high, which is a significant limitation of this method. Previous studies have indicated that 40%–80% of patients with paroxysmal AF experience a 1‐year period free of recurrent arrhythmias.^3^, ^4^, ^5^, ^6^, ^7^ The effectiveness of the 12‐month AF‐free period was significantly improved when using the CLOSE protocol, where it was 94% compared to 80% in patients using conventional contact force guided PVI.^8^ The CLOSE protocol considers the Ablation Index (AI) parameter, which is a function that takes into account the radiofrequency (RF) application power, contact force, and duration of the application. AI can only be used when using the point‐by‐point method. Some operators, however, apply a dragging technique involving continuous application and gradual shifting of the catheter to achieve an ablative line. Although both methods allow PVI to be obtained, looking at the results of recent studies, it seems that the point‐by‐point method—especially with the use of AI—allows more permanent changes to be made which results in a reduction in the frequency of AF recurrences.

RF ablation is associated with intentional damage to the myocardium to achieve PVI.^9^ It seems that the size of the damage to the muscular tissue of the heart affects the magnitude of the inflammatory response, which may affect the rate of recurrence of arrhythmias.^10^ In addition, numerous applications, especially those that do not cause transmural damage to the myocardium, may increase the risk of atrial flutter—an arrhythmia observed in some patients after PVI.^11^

The aim of the presented study is to compare the amount of damaged myocardial tissue, assessed by the serum level cardiac troponin I (cTnI), among patients after PVI performed point‐by‐point (CLOSE protocol) and dragging technique.

## 2 METHODS

The current study is the subanalysis of the ACTIVE‐AF (Inflammatory Response as a Prognostic Factor of Recurrence of Atrial Fibrillation After Ablation) trial (NCT02844959). The ACTIVE‐AF study was designed to evaluate inflammatory response as a prognostic factor of AF recurrence after ablation. Patients ≥ 18 years old with paroxysmal AF qualified for radiofrequency PVI procedure were included in the study. Exclusion criteria were as follows: previous PVI procedure, planned ablation other than PVI during the procedure, AF at the beginning of ablation procedure, direct current cardioversion during the procedure or in 24 hours after the procedure, usage of “single shot” devices. All patients underwent 7‐day Holter monitoring directly after the PVI was performed. Blood samples were collected twice: right before the ablation—taken from the vascular sheath in the femoral vein and 24 hours after the end of the PVI procedure. Study was registered on clinicaltrials.gov (NCT02844959). All patients signed informed consent for participation in the trial. The study protocol was approved by the local Ethics Board (number KB/143/2016).

### 2.1 Pulmonary veins isolation procedure

**FIGURE 1:**
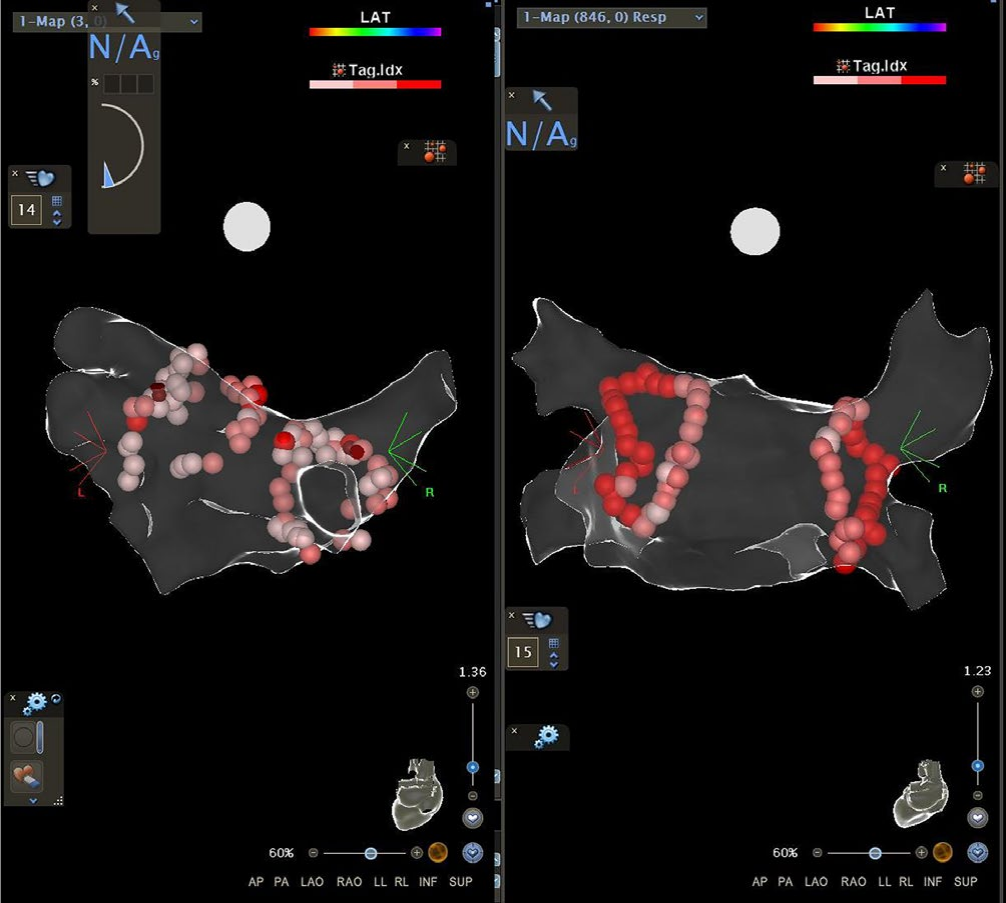
Sample CARTO map visualizations showing dragging and point‐by‐point methods in the posterior‐anterior projection

**FIGURE 2:**
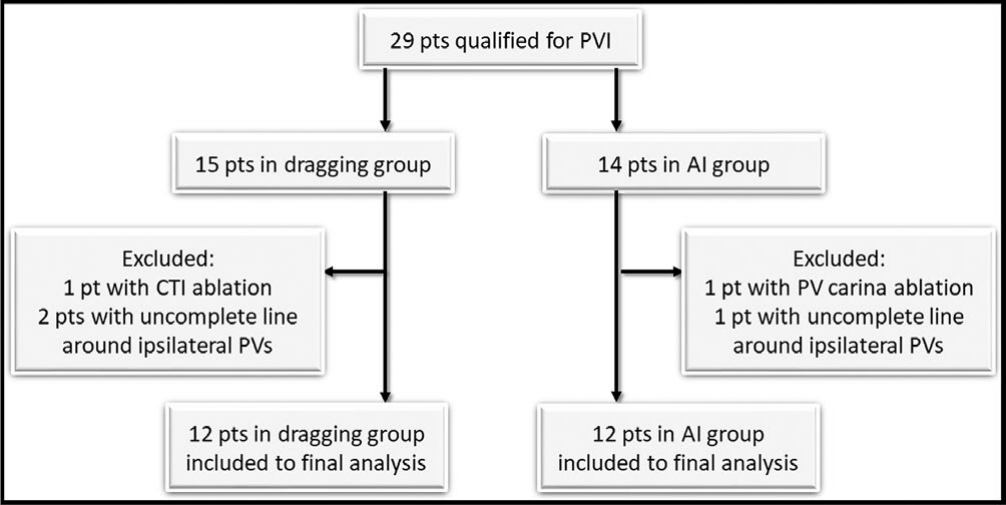
Flowchart of patients’ selection to final analysis. (AI – Ablation Index; CTI – cavo‐tricuspid isthmus; pt – patient; PV – pulmonary vein; PVI – pulmonary vein isolation)

All procedures were performed, between July 2016 and August 2018, as wide area circumferential ablation with paired isolation of ipsilateral pulmonary veins. During the procedures ThermoCool SmartTouch SF® and 20‐pole Nav Lasso catheters (Biosence Webster) were used. Fast anatomic map of left atrium was created for every patient. All PVIs were performed by the same operator with CARTO 3D mapping system (Biosence Webster). In the first group PVI was performed with dragging technique using contact force range between 5 and 40 g. In the second group AI protocol was used with AI value of 400 in posterior wall and 550 in other parts of the ablation line.^8^ In both groups energy of 25 W was used during applications in the posterior wall and 30 W in the other regions during PVI. CARTO map visualizations with visualized both dragging and point‐by‐point methods are presented in Figure 1. Acute PVI was defined when at least entrance block was confirmed with Lasso catheter or pacing maneuvers. The effect of the ablation was reassessed 20 minutes after the last application. In case of conduction recurrence additional applications were made. To select homogenous group of patients, procedures with additional applications (other than lines around the ipsilateral pulmonary veins) were excluded from the final analysis (Figure 2).

From July 2016 to March 2017 all PVI procedures were performed with dragging technique. In that time data of 15 patients were gathered and 12 patients were included in the final analysis. From September 2017 CLOSE protocol was implemented to PVI procedures. To make the comparison between groups, the equivalent number of patients from the AI group was included in the final analysis.

### 2.2 Follow‐up

In every patient after PVI procedure four 24‐hours Holter ECG tests at 3‐months intervals were recommended to perform. Patients’ follow‐up was conducted based on Holter ECG test results and a telephone conversation. Arrhythmia recurrence diagnosis was based on a minimum 30‐seconds episode of AF, atrial flutter or atrial tachycardia recorded by Holter ECG or one of these arrhythmias recorded by 12‐lead ECG.

### 2.3 Laboratory assessment

All laboratory assessments were performed from venous blood samples. Troponin I level was assessed directly before the procedure and 24 hours after the end of procedure. All other parameters including compete blood count, ions, creatinine, and liver enzymes were assessed from blood samples collected in the 24 hours before the procedure. In the following analysis, there was lack of baseline cTnI assessment in one patient in dragging group and in two patients in AI group. These patients were excluded from the analysis assessing cTnI changes. All laboratory assessments were performed directly after the blood sample collection. To assess cTnI level Siemens Dimension EXL automatic chemiluminescence immunoassay analyzer was used. According to the reagent manufacturer's documentation, normal values of cTnI were considered as 0.00‐0.056 ng/mL.

### 2.4 Statistical analysis

Distributions of continuous variables were calculated with Shapiro‐Wilk test. For all continuous variables normal distribution could not be excluded. All continuous variables were presented as median value and standard deviation (SD). For categorical variables percentages and number of subjects were used. Differences between groups were assessed with Fisher's exact test and Student's *t*‐test, for categorical and continuous variables, respectively. Relationships between continuous variables were calculated using Pearson's correlation. Analysis of arrhythmia recurrences was based on Kaplan‐Meier curve. Tests were considered significant for *P* < .05. Knowing the mean values of cTnI level and standard deviation in dragging group assumed that for 70% power of the test, analysis would be statistically significant for cTnI blood level <1.9 ng/mL in AI group.^12^ Statistical analysis was calculated using SAS® software (Cary, NC, USA), version 9.4.

## 3 RESULTS

Of the 29 patients screened for the presented study, 24 were included for the final analysis, 18 (75%) male and 6 (25%) female. The mean age of patients was 59.3 ± 7.7 years. There were no differences in the clinical characteristics between both groups. A lower contact force during RF applications (11.5 ± 1.7 vs. 13.3 ± 2.1; *P* = .04) and trend in shorter total x‐ray time (8.6 ± 5.4 vs. 5.3 ± 3.2 min.; *P* = .093) was observed in the AI group compared with the standard group with no differences in total procedure time (146.3 ± 28.9 vs. 153.2 ± 37.1 min.; *P* = .62) and total RF applications time (48.3 ± 8.4 vs. 45.6 ± 7.7 min; *P* = .43). A full comparison of both groups is presented in Table 1.

**TABLE 1:** Patients’ characteristics. (cTnI – cardiac Troponin I; RF – radiofrequency)

	Dragging group (n = 12)	Ablation index group (n = 12)	*P* value
Demography			
Age	57.7 ± 9.4	61.0 ± 5.4	0.30
Male	83%	67%	0.64
Clinical characteristics			
CHA_2_DS_2_VASc score	1.25 ± 0.97	1.75 ± 0.97	0.22
Hypertension	58%	67%	1.00
Dyslipidemia	33%	33%	1.00
Diabetes mellitus	17%	9.1%	1.00
Heart failure	8.3%	8.3%	1.00
History of stroke	0.0%	8.3%	1.00
Vascular disease	0.0%	17%	0.48
Laboratory findings			
White blood cells [1000/µL]	7.1 ± 1.5	8.0 ± 2.0	0.24
Hemoglobin [g/dL]	14.7 ± 1.0	13.6 ± 1.3	0.44
Estimated glomerular filtration rate [mL/min/1.73 m^2^]	73.3 ± 16.7	62.9 ± 12.3	0.11
Aspartate transaminase [U/L]	28.4 ± 16.0	25.8 ± 10.5	0.65
Alanine transaminase [U/L]	39.9 ± 21.3	30.2 ± 12.7	0.20
Thyroid‐stimulating hormone [mU/L]	1.45 ± 0.68	1.62 ± 1.2	0.68
cTnI level before the procedure [ng/mL]	0.015 ± 0.032	0.004 ± 0.006	0.30
cTnI level 24 h after the procedure [ng/mL]	3.37 ± 1.82	1.98 ± 0.64	0.03
Ablation procedure			
Procedure time [min]	153 ± 37	146 ± 29	0.62
RF ablation time [min]	45.6 ± 7.7	48.3 ± 8.4	0.43
Number of RF applications	58.4 ± 27.8	78.1 ± 18.1	0.06
Mean contact force [g]	13.3 ± 2.1	11.5 ± 1.7	0.04
X‐ray time [min]	8.6 ± 5.4	5.3 ± 3.2	0.09

### 3.1 Assessment of myocardial necrosis

**FIGURE 3:**
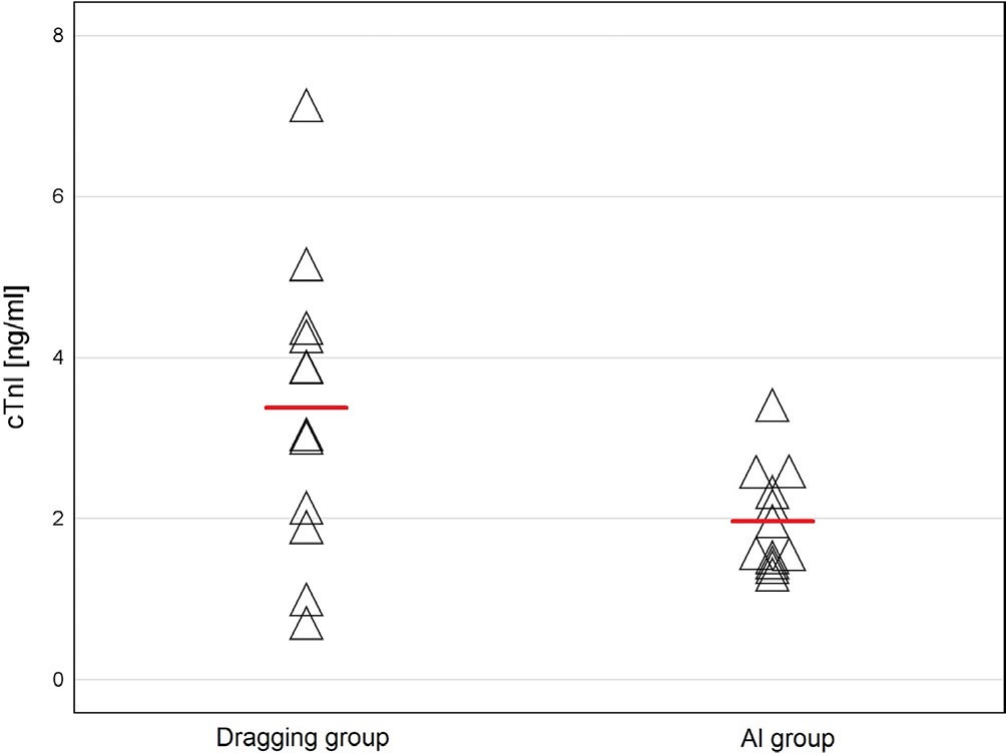
Cardiac Troponin I level 24 h after pulmonary vein isolation. Horizontal lines show mean values. (AI – Ablation Index; cTnI – cardiac Troponin I)

Twenty‐four hours after the PVI procedure cTnI levels were significantly lower in the AI group than in the dragging group (1.984 ± 0.644 vs. 3.369 ± 1.818 ng/mL; *P* = .026) (Figure 3), with no difference in mean baseline cTnI level (0.004 ± 0.006 vs. 0.015 ± 0.032 ng/mL; *P* = .304). Differences between pre‐ and postprocedural cTnI levels also differed significantly between the AI and dragging groups (1.926 ± 0.648 vs. 3.291 ± 1.972 ng/mL; *P* = .042).

**FIGURE 4:**
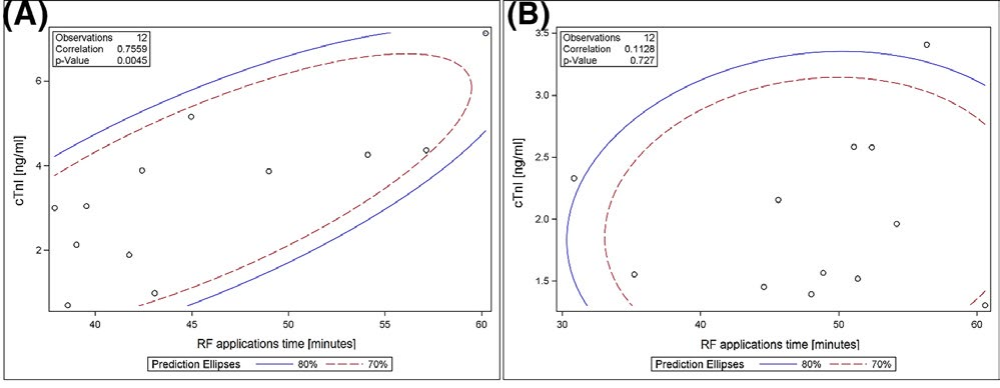
Correlation between radiofrequency applications time and cardiac Troponin I level assessed 24 h after pulmonary vein isolation in dragging group (A) and Ablation Index group (B). (cTnI – cardiac Troponin I; RF – radiofrequency)

**FIGURE 5:**
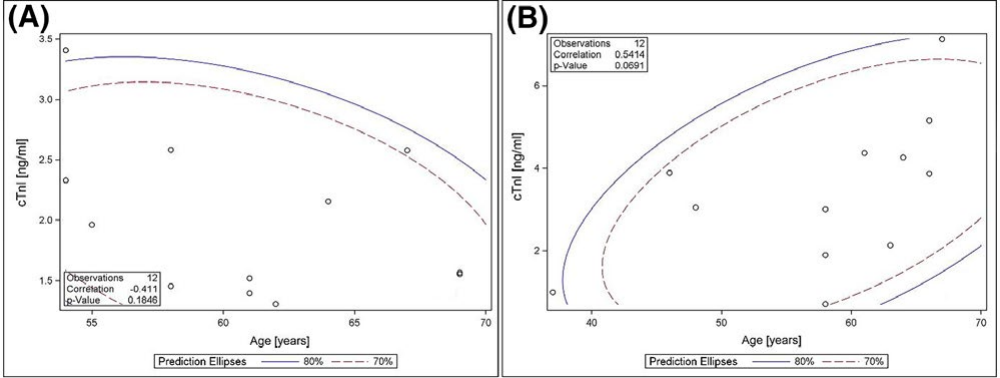
Correlation patients’ age and cardiac Troponin I level assessed 24 h after pulmonary vein isolation in dragging group (A) and Ablation Index group (B). (cTnI – cardiac Troponin I)

CTnI levels 24 hours after PVI procedure positively correlated with total RF application time in the dragging group (*r* = .76; *P* = .005). While, this relationship was not observed in the AI group (*r* = .06; *P* = .81) (Figure 4). Similarly, a trend was observed in the dragging group between cTnI levels 24 hours after PVI and age (*r* = .54; *P* = .07) without correlation between these two variables in the AI group (*r* = −.41; *P* = .18) (Figure 5).

### 3.2 Arrhythmia recurrences

**FIGURE 6:**
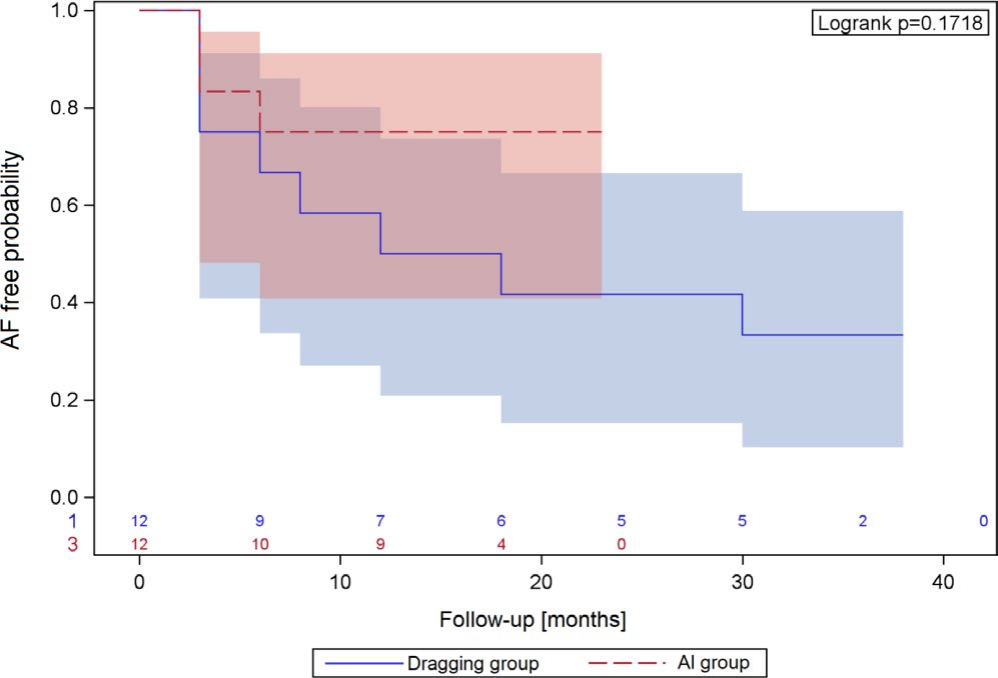
Arrhythmia recurrences over time in patients in the dragging group and Ablation Index (AI) group

Follow‐up of the patients after indexed PVI procedure was performed after median time of 33 months in the dragging group and 16 months in the AI group. Analysis of arrhythmia recurrences revealed no differences between the groups (*P* = .17). Kaplan‐Meier curve of arrhythmia recurrences is presented in Figure 6.

## 4 DISCUSSION

The presented study showed that PVI with CLOSE protocol using AI results in a reduction of myocardial necrosis assessed with serum cTnI level compared to the dragging technique. A positive correlation between total RF time and serum cTnI level was revealed only in the dragging group.

The increase in the concentration of markers of myocardial necrosis after RF ablation was confirmed in previously published studies. Haegeli et al showed that 4 hours from the end of ablation, cTnT was elevated and its value exceeded the cut‐off point for the diagnosis of acute myocardial infarction (AMI).^13^ Interesting results were also obtained in the study evaluating the stability of biomarkers of myocardial necrosis and comparing the concentrations of these biomarkers between patients who underwent PVI with RF and who underwent PVI with balloon cryoablation.^14^ The first important finding in this study was the high stability of cTnI molecule at both low and high temperatures. This means that the high temperature reached during RF application should not have a significant effect on the obtained results of cTnI level. Secondly, this study showed that an increase in cTnI concentration above the cut‐off level for AMI had already occurred in the first hour after the end of the procedure. It is worth bearing in mind, however, that the start time of an RF application is on average >1 hour before the end of the procedure.

It has been shown that in case of acute AMI, the peak cTnI concentration is reached after 18‐24 hours of symptoms onset and an increase above the upper limit of the norm may persist for up to 14 days.^15^ Results of studies describing the effect of troponin on the size of the myocardial necrosis are not conclusive.^16^ A study by Chia et al analyzing serial cTnI serum concentration assessments in patients after AMI treated with primary coronary angioplasty, showed the best correlation between cTnI concentration and the size of cardiac necrosis for blood samples collected ≥24 hours after onset of ischemia.^17^ Bearing in mind the variable total time of RF applications in the analyzed patients, it seems that blood samples collected 24 hours after the end of the procedure allow determination of the peak cTnI concentration, and at the same time it can be a reliable predictor of the size of ischemia. This assumption is confirmed by a work assessing the dynamics of troponin concentration and other markers of ischemia and inflammation in a group of patients undergoing RF ablation.^18^ In this study, peak cTnT values were demonstrated on the first day after surgery, followed by a downward trend in the following days. Analogous concentration dynamics were observed for creatine kinase muscle‐brain fraction (CKMB). Similar results were demonstrated by Wójcik et al, following serial measurements within 1‐24 hours it was shown that the peak concentrations of cTnI occurred 24 hours after the end of PVI.^14^ Interestingly, the median maximum cTnI concentration in the RF group was 1.29 ng/mL, therefore it was lower than the values obtained in the current study, both in the dragging group and the AI group. The lack of a precise description of the methodology of RF procedures in the cited work does not allow identification of factors that could be the source of the difference.

In their study describing the CLOSE protocol, Philips et al showed a significantly shorter procedure time and shorter time of RF application among patients using the CLOSE protocol compared to the previous method using only contact force, a similar difference was not observed in the presented study.^8^ Despite the mean procedure time being comparable to the Philips study, the application time was around 35% longer in the current study/analysis, this may be the result of the operator's learning curve and the technique change from dragging to point‐by‐point. Despite the lack of time difference between the dragging and AI groups in RF application, the difference in cTnI concentrations indicates more targeted application performance, it also means that the difference in cTnI levels should not be explained by the application time, but rather by the stability of the catheter. Interestingly, contact force was slightly higher in dragging group compared to AI group. However, this difference seemed to be not sufficient to justify significantly higher cTnI levels in AI group. Particularly, the positive correlation between total ablation time and cTnI levels 24 hours after the end of the procedure was interesting. Haegeli et al did not show a significant difference between the number and total time of RF applications and the cTnI levels measured 4 hours after the end of the application.^13^ Contrary results were obtained in the study assessing the degree of myocardial damage during PVI using RF ablation and balloon cryoablation, this study showed a strong correlation between the total RF application time and the cTnI serum levels evaluated 6 hours after the end of the procedure.^19^ An analogous relationship was demonstrated in the presented analysis only in the dragging group. The lack of such correlation in the AI groups may again indicate more targeted changes and greater stability of the ablation catheter position.

Outcome of the patients after the PVI differed between the groups, however, it did not reach statistical significance. Small number of patients in both groups seems to be an important limitation in comparison of the procedure effectiveness. In presented study, in the AI group effectiveness of single PVI procedure was lower than described in Phlips et al publication.^8^

## 5 CONCLUSIONS

The presented study revealed that compared with standard, continuous applications (dragging technique), CLOSE protocol implementation results in a reduction of myocardial injury during catheter PVI in patients with paroxysmal AF. Moreover, when using CLOSE protocol, myocardial injury is independent of RF application time.

## DISCLOSURE

The local institutional review board of Medical University of Warsaw approved trial on October 6, 2016 (number KB/143/2016), and the trial was registered at the ClinicalTrials.gov on July 26, 2016 (identifier: NCT02844959). All the other authors report that they have no relationships to disclose that are relevant to the contents of this paper.
